# Advancing South-South cooperation in education: Indonesian experience with South Africa

**DOI:** 10.12688/f1000research.123311.2

**Published:** 2023-02-20

**Authors:** R. Dudy Heryadi, Shanti Darmastuti, Ayu Anastasya Rachman

**Affiliations:** 1 Padjadjaran University, Indonesia, Bandung, Indonesia; 2 Universitas Pembangunan Nasional Veteran, Jakarta, Indonesia; 3 Universitas Bina Mandiri, Gorontalo, Indonesia

**Keywords:** South-South Cooperation, Middle-Income Countries, Indonesia, and South Africa Education Cooperation

## Abstract

Education collaboration is important to increase the quality of human capital. South-South cooperation is conducted to exchange resources, technology, skills, and knowledge between southern countries. The purpose of this research is to investigate the collaboration between Indonesia and South Africa toward advancing South-South Cooperation in education. The data collection used in this qualitative research is an interview and literature study with 9 purposive informants from the Indonesian Ministry of Foreign Affairs, the Indonesia Ministry of Education and Culture, and the Embassy of South Africa. The data were obtained within six months. The interview was primarily used to explore the roles and practices of government institutions and universities in fostering education collaboration and human resources development. In this study, the South-South Cooperation helps Indonesia address its educational needs. The result showed that most international education cooperation between both countries is agreed upon at the University-to-University level through a Memorandum of Understanding (MoU), facing less sustainable challenges and low impact. Therefore, an investment-led model, multi-stakeholder collaboration, and a tool to monitor and evaluate this process are needed to improve South-South Cooperation to achieve its goals.

## Introduction

The international development landscape has shifted and allowed new forms of cooperation to flourish. International cooperation has always promoted sharing information and best practices as significant features in education. Compared to the North-South Cooperation, the South-South is perceived as more economical, effective, and favorable. Therefore, this research seeks to fill the gap in the education cooperation model between Indonesia and South Africa to maintain diplomatic relations between the two countries.

The South-South Cooperation (SSC) establishes a collaborative scheme among the least or Middle-Income Countries, most of which are located in the southern part of the globe. In practice, it incorporates the basic principles of relationships between sovereign states, such as respect for sovereignty, non-interference in internal affairs, and equal rights. This process promotes the exchange of successful experiences between countries that share the same historical realities with similar challenges. Indonesia has been working closely with southern countries since the Asia-Africa Conference, the Non-aligned Movement (NAM) formation, and the G-77 in 1955, 1961, and 1964. Today, it is committed to enhancing its role and contribution to the development of South-South Cooperation, especially in South Africa.

Education is important in most developing countries because it aids in the formation of human capital for long-term investment and nation-building. Countries with a focus on human capital become more attractive to domestic and foreign private investment ( Becker *et al.*, 2001). Many universities in developing countries carry out forms of collaboration with universities from other country to create international quality education. The collaboration usually related to joint publications, development of joint teaching materials, and joint coordination to obtain international funding. Several challenges are faced by developing countries such as the inability of students in terms of funding for student exchange programs in developed countries. In addition, limited educational infrastructure, such as research infrastructure, is also a separate obstacle ( Nguyen *et al.*, 2015).

Partnership is one way to develop capacity both in the fields of research and education ( Hanada, 2021). However, to improve the implementation, it is necessary to have a clear form of partnership between the actors involved, an evaluation of the partnerships that are being carried out, as well as the development of knowledge and skills ( Zhu & Chikwa, 2021). Partnerships based on mutuality can create mutual benefits. This can happen because the partnership was designed jointly by the universities from both parties. This is different from aid-based partnerships where the scheme tends to have been designed in advance, such as who will receive assistance and the form of the scheme.

Through the Ministry of Education and Culture, Indonesia has carried out systemic reforms by revising various regulations, including the national education law on curriculum, research, expertise, and accreditation. Indonesia’s education system endorses the philosophy of “ *Merdeka Belajar*,” translated as freedom of learning. This concept focuses on revising the curriculum of higher institutions towards greater quality, competence, and expertise of teachers and students. It is also aimed to advocate the academic mobility of students and lecturers outside their study programs, including cross-university and continents.

The presence of various forums such as the Indonesia-Africa Infrastructure Dialogue (IAID) and the Indonesia Africa Forum (IAF) help the two countries to meet each other’s needs. South Africa is Indonesia’s largest trading partner in Africa therefore, the increase in capital goods procurement from this country indicates productivity, which is mainly related to the need for infrastructure development. Indonesia has significant potential to increase the economic balance of South Africa. Both countries share scientific expertise and scholarly exchanges, which are largely unprompted by the government. Although this collaboration has been mentioned multiple times in forums, no cooperation framework has been specifically designed, implemented, or evaluated for educational-related programs such as scholarship and exchanges. The progress seems sporadic and unmonitored, which led to the inception of this research.

According to the Indonesian Ministry of Foreign Affairs hosting “Diplomatic Training/Education “for African diplomats, the educational activity at the state level is technical vocation and training. Meanwhile, at the non-state level, it is carried out through different forms, including the signing of a Memorandum of Understanding (MoU) in universities from both countries, the establishment of the African Indonesian Center of Studies in Indonesian University, scholarship scheme for African students and by sending speakers to international seminars. A study conducted by McLaughlin (1996) provides a framework for global education by stating that it builds cross-cultural understanding and develops the cooperative attitude needed to solve world problems.

From several studies discussing educational cooperation, existing research does not yet specifically explain educational cooperation within the framework of SSC. Therefore, this research will specifically discuss the educational cooperation between Indonesia and South Africa within the framework of south-south cooperation. The existing SSC between Indonesia and South Africa is mostly in the agriculture sector. However, satisfaction would still be lagging, without the sustainability of education cooperation, especially in the important agenda, such as teacher’s, girls and women’s education, technical vocation and training, as well as education on technology and halal products. Therefore, this study aims to analyze the existing model of education funding and cooperation between southern countries and later on construct the revised model of SSC in education between Indonesia and South Africa

## Literature review

### Education as human capital

According to the literature, education can generate human capital and contribute to economic growth and development by creating human capital. The notion of education as a form of human capital was first used in the study of Schultz (1963), sometime after he discussed whether or not knowledge and skills become a sort of capital ( Schultz, 1961). A focused study also conducted by Becker between 1960 and 1964 theorized the linkage between economic development meaning high-income revenue and education. Similarly, earlier studies had emphasized the role of education as an intangible capital which means “improvement in basic science, technology, business administration, education and training” to enhance the productivity of a nation ( Mincer, 1958; Fabricant, 1959; Schultz, 1961), even further they have found the direct relationship between education and knowledge of the population and national growth, estimated “that knowledge and education accounted for at least 43% of national income growth” and suggested “education contribute to health and nutritional improvements” ( Denison, 1962, 1974; Schultz, 1963; ). The human capital theorized the type of education and knowledge obtained from both formal education, informal education at home and work, vocational education, and on-the-job training ( Cohn & Geske, 1990; Schultz, 1981; Corazzini, 1967; Mincer, 1974). Despite the breakthrough, education as human capital receives backlash by criticism of the quality of education, impact of learning on experience, the role of nutrition and health, and unemployment among high credentials graduates ( Wößmann, 2003; Gundlach, 1997; Livingstone, 1997).

In contemporary studies, more attention was paid to adult education, especially the knowledge and competence required in the labor market. Countries particularly developing have included human capital development in their national planning ( Devadas, 2015). A study by Miyamoto (2003) found that some developing countries have not yet achieved access to education, quality of education, and adult literacy. The difficulties had prompted the policy initiative namely ‘education for all’ in collaboration with international donors, inter-government, and NGOs. Miyamoto (2003) also found that human capital can contribute to civil liberties, political stability, health, and reduced crime and corruption which are determinants of Foreign Direct Investment (FDI). Muhr (2016) explains that not only security and economy, but human development is also a shared responsibility of countries, “ *commitment to mutual support and joint efforts to achieve sustainable and integral human development, and the appropriate care of developing countries*”. The position of education in developing major countries is then adapted to various global sustainable development agendas. The MDGs and SDGs emphasize achieving universal, free, and compulsory primary education through Education for All (EFA). Improving and enhancing the quality of education and gender equality to support long-life learning is the collective responsibility of countries. McLaughlin (1996) provides a framework for global education, “ *global education seeks to teach people how to live in an increasingly interconnected and interdependent world*”. This educational method serves to build cross-cultural understanding and develop the cooperative attitude needed to solve world problems. The emergence of developing or middle-class countries as new donors and providers of technical assistance in the 2000s has reshaped the landscape of international development cooperation. This cooperation allows countries to respect each other’s cultures and develop relations and friendships. While building various ideas and strategies to deal with global issues and various perspectives.

### SSC in education

The concept of SSC is understood as *“the best practice transfer and the exchange of resources, such as but not limited to technology, skills and knowledge among the members of southern countries, as well as promoting the development of social, economic, cultural, political by building coalitions.*” This cooperation model combines funding, knowledge, expertise, and technology to transfer between Southern countries, either bilaterally or multilaterally. A previous study on SSC between Ethiopia, Kenya, and Uganda shows that South-South Cooperation maintains domestic stability covering various fields, such as Agriculture, Trade, and Education. Siahaan (2017) also found that SSC is an effective form of activity to increase “friends,” known as diplomatic relations. Important to note is evidence which support the output of the best practices transfer and programs is infrequent and it is required to pay more attention to evaluate these practices and programs ( du Toit *et al.*, 2017)

The emergence of developing or middle-class countries as prospectus donors and providers of technical assistance in the 2000s has reshaped the landscape of international development cooperation. This process allows countries to respect each other’s cultures and develop friendships while building ideas and strategies to deal with international issues. International educational cooperation has monetary and non-monetary significant effects on countries. In monetary terms, the higher a person’s education level, the lower their unemployment and poverty rates with better income returns, used to build the country through taxes. Meanwhile, in non-monetary terms, education affects a person’s health and nutrition practices, childcare, and participation in social activities.

Regarding the form of educational cooperation, one form of educational cooperation can be found in the case of cooperation between China and Kenya. In this matter, China provides a form of training to strengthen the capacity of Kenyan human resources. In addition, the cooperation between the two sides appears in the form of providing scholarships from both the Kenyan and Chinese governments to Chinese students studying in Kenya and vice versa. The educational cooperation carried out between the two parties is carried out in a reciprocal South-South mode of cooperation. The interesting thing here is that China also provides this form of training through Chinese companies operating in Kenya, and the trainers are not only from China but also from Kenyans themselves ( King, 2010). The scholarship form is also one of the programs used by the United States in conducting educational cooperation with Africa. Scholarships are awarded in both technical and social sciences at US educational institutions ( Amanor 2013).

Cheru (2016) explained the cooperation between Ethiopia and India in the field of education. The form of educational partnership carried out by the Indian government provides 50 scholarships for students from Ethiopia to study at universities in India every year. This exchange is monitored by a shared working group which was founded in 2007. Education is one of India’s grant priority sectors. The grant program is given a priority on capacity building in the field of education. Several education programs can be seen in the construction of schools (in the Maldives) and assistance in transforming the South African education system ( Tilak, 2016). From several forms of educational cooperation within the framework of SSC, it can be seen that the provision of scholarships and the provision of training in the context of capacity building are the main programs in educational cooperation.

Some studies have discussed international aid to education, which also helped the exploration of the SSC model. In general, there are two kinds of funding models in international education: the traditional development-aid model and the southern model. The traditional development aid was given by INGOs, such as but not limited to, Development Assistance Committee (DAC), OPEC and OECD countries. Whereas the southern model is given by Non-DAC, Non-OPEC, or “emerging donors” ( du Toit *et al.*, 2017; Zimmerman & Smith, 2011; Manning 2006). The latter one is therefore in line with the SSC model comprising the collaboration between partners in the global south which pay a greater focus on partnerships and cooperation. In terms of cooperative education programs based on SSC, there are two kinds of relationships. The first one is vertical intervention by the foreign government in local society. The second one is a twinning relationship or triangular model, which also means best practice transfer ( Walensky & Kuritzkes, 2010; Sa e Silva, 2009). Other than funding, Dr. Diplomat in The Economist (2007) mentioned a study about the education cooperation model between Cuba and Africa which was implemented as capacity building and the provision of infrastructure.

There is a discussion surrounding the effectiveness of various implementation models of the aid, as well as the measurement of impact in the recipient country and population. There is so much theory about the intended outcomes of development, rather than what aid micro-aims to achieve. The focus of the conversation is on how aid is utilized to enhance the quality of life rather than economic return alone ( UNDP, 2010). Some suggest the need to comprehend the motivation of all the actors involved in the aid relationship, from the political desires and to the consequence of the relationship of donor and recipient that were formed historically ( Schraeder PJ, *et al.*, 1998). Furthermore, Rosseel *et al.* (2009) express the social role of higher education in development. University has a prominent role in promoting and conducting capacity building such as training and workshops that are beneficial to communities, both in which they are based and beyond. As the co-creators of knowledge, universities promote society participation and produce types of science that address the needs of society.

Previous models may have not been focused on the process of monitoring and evaluating the programs, and rather on theorizing so much of the intended outcomes of the development ( Du Toit, 2017). Scholars that discussed SSC have just focused on limited types of education cooperation such as scholarship, training, and workshop, and less attention has been paid to more progressive models, like investment-led mode. Therefore, our model these two factors have been emphasized to fill the gaps.

## Methods

This research is qualitative research, specifically case study. According to Creswell & Cresswell (2018), it is explained that a case study form is a research design in which the researcher develops an in-depth analysis of a topic. The cases taken are limited by time and activity, and the researcher collects detailed information using various data collection procedures over a continuous-time. Analysis of the data used in this study is divided into three stages, namely data reduction, data display, and verification. As explained by Miles and Huberman (1994), data reduction focuses on the process of selecting, focusing, abstracting, and transforming the data that appears in the transcription. In this case, data reduction is based on the framework of thought and research questions that have been determined. Next, the data display stage will be carried out. The results of the data reduction carried out will be made in the form of tables, or matrices to organize the reduced data. The last is the data verification process and making conclusions on the data that has been obtained.

### Study design

This research uses qualitative methods to unravel the SSC in Education between Indonesia and South Africa. The qualitative method was selected because the topic requires an understanding of how the education cooperation process is carried out and developed in the future. The primary concepts in this study are Education as Human Capital and South-South Cooperation Model in Education.

### Participant selection

The purposive sampling method was used during the interviewing process to acquire information-rich cases related to education activity between Indonesia and South Africa. The total number of informants is nine people, three per institution. The informants’ criterion is based on two points. First, having the expertise and the task to coordinate foreign cooperation specifically with southern countries. Second, his/her position is exclusive as the director and/or in charge of the public relations in the relevant institution as follows: the Indonesia Directorate General of Higher Education, the Indonesia Ministry of Foreign Affairs, and the South Africa Embassy in Indonesia.

### Data collection

Data were obtained from primary and secondary sources with the duration of data collection being six months covering the data of SSC between Indonesia and South Africa from 2015 to 2020. The primary data were collected through an interview process with selected informants, and the secondary data were from documentation studies, such as ministerial regulations, technical guidelines, scientific journals, and mass/electronic media, in the forms of research reports, documents, archives, or videos. The data set was established to collect the intended information about SSC in education. Firstly, the information regarding the SSC Indonesia with South Africa which consist of: a) programs; b) agreement; c) implementation, was collected from the Indonesia Ministry of Foreign Affairs (code of Informant 2) and The South Africa Embassy in Indonesia (code of Informant 3). Secondly, the data about Indonesian scholarship, exchange programs, and capacity building with South Africa were obtained from the Indonesia Directorate General of Higher Education (code of informant 1)

### Data analysis

This research is deductive, focusing on identifying existing educational cooperation between Indonesia and South Africa and analyzing the gap using the South-South Cooperation Framework. In this study, Taguette open-source was used to help with qualitative data analysis in different stages. In maintaining validity, a triangulation method was formed using various available data sources, which were juxtaposed to select the valid ones. Triangulation is a technique which uses multiple methods and/or data sources in a qualitative approach to construct an holistic understanding of phenomena ( Patton, 2002). Meanwhile, this research triangulation method matches the data obtained through the interview and literature study methods.

### Ethical approval

All informants completed the consent form. It was made clear that their identities and response would remain confidential and only used for the data analysis of this study. Participants were voluntary, meaning that they could decide to withdraw from any questions they felt not comfortable with. Permission to record the data was obtained from informants through email correspondence to ensure the accuracy of the analysis. This study protocol was approved by the ethics committee from the research, education, and society outreach department of UPN Jakarta.

### Findings

The results of the interview show that increasing the competence and capacity of human resources is one of the main objectives in the implementation of south-south cooperation. This capacity building can be carried out by sending human resources to internship or training activities. As stated by an informant from the Ministry of Education and Culture of the Republic of Indonesia who explained that:

“In creating quality human resources under the direction of President Joko Widodo, the position of international cooperation in the field of Indonesian education is very diverse. One of these things can be seen through bilateral cooperation, among others: the existence of cooperation to improve the quality of learning in terms of encouraging and facilitating the exchange of information, and scientific publications on early, basic, secondary, vocational, and technical education through formal, non-formal and informal education” [Informant 1.2, Table 1]

**Table 1.** Education Cooperation with South Africa as The Development Agenda (Human Capital).

No	Informants	Point of Concerns
1	Informant 1.1.	One form of capacity building in Indonesia-South Africa cooperation is through training and diplomatic education between the two countries
2	Informant 1.2.	One of the goals of educational cooperation is to improve the quality of human resources
3	Informant 1.3.	Educational cooperation is needed to create human resources following the demands of technological development. Capacity building of human resources not only in academic but also non-academic fields
4	Informant 2.1.	One form of capacity building for human resources in cooperation with a country in Africa is the form of sending education personnel to do internships in partner countries.
5	Informant 3.2.	Capacity building of human resources is the foundation for educational cooperation
6	Informant 3.3.	Good relations between Indonesia and South Africa, especially in the South-South Cooperation, can provide benefits to both countries

Both countries’ education and skills development are critical because they share similar challenges such as poverty, teacher’s education, girls’ education, adaptive curriculum, and technological disruption. In 2063 African Union Agenda conveyed the need for a revolution in education and skills, which was emphasized in the Common African Position (CAP) of the Sustainable Development Goals (SDGs). According to CAP, there is a need to promote and support research, technology, and innovation *,* to build human resources in Africa. This includes establishing education centers, an African accreditation board to monitor the educational standards, and strengthening the relationship of Pan African University.

Meanwhile, the educational philosophy of “Merdeka Belajar,” applied at the tertiary level as Strategic Planning of Indonesia Education Development to accelerate the quality, is categorized into three different streams. The first is the provision of scholarship programs for lectures, followed by organizing research collaboration programs, visiting professors, student exchanges, joint laboratories, and others. The last is the 3) fulfillment and utilization of university infrastructure.

Various forums, such as the Indonesia-Africa Infrastructure Dialogue (IAID) and the Indonesia Africa Forum (IAF), help the two countries meet their needs, especially trading partners. Despite the significance, the education agenda has not been discussed much bilaterally between Indonesia and South Africa. This is because the investment-led model is a suitable cooperation mechanism between the two countries. Therefore, to develop mutual respect toward freedom and democracy, a business-oriented approach to cooperation is needed, which allows to achieve win-win and avoid interference in internal affairs. On the contrary, the unconditional cooperation of the SSC may entrench the unaccountable political elites as a trade-off of democratic reforms and jeopardize the protection of human rights.

### Indonesia’s contribution

Indonesia’s role as a contributor under the framework of SSC has been initiated since 1950, and it is increasingly significant, especially in education.

*The Indonesian government realizes that higher education plays a role to help the country achieve socio-economic goals and have practical impact on society. Higher education promotes responsible citizens, which hold ethical behavior, educational desires, professionalism in various fields, and cross-border engagement. Therefore, as a part of our commitment in helping the international public to survive the global challenges, sharing our experience and resources by nurturing a highly skilled and educated society has also become our priority.* [Informant 2.2, Table 2]

**Table 2.** Education cooperation existing model between Indonesia and South Africa.

No	Informants	Point of Concerns
1	Informant 1.1.	Increase scholarship quota
2	Informant 1.2.	Scholarships are given, such as scholarships to study Indonesian Language and Indonesian Cultural Arts
3	Informant 1.3.	The scholarship program aims to promote Indonesian culture and language among the younger generation, as well as strengthen cultural ties among students from various cultural backgrounds.
4	Informant 2.2.	There is a Memorandum of Understanding between the Government of the Republic of Indonesia and the Government of the Republic of South Africa regarding Cultural Cooperation
5	Informant 2.3.	South Africa is the only African country that has a strategic partnership agreement with Indonesia
6	Informant 3.1.	Cooperation between universities in Indonesia and universities in South Africa

According to Aikins (2008), the socio-political and economic crisis in the African region in the 1970s and 1980s has reduced funding for research and academics, scholarships, decline in student enrollment, and problems in academic-related tasks. Other challenges are related to shortages of a qualified labor force and an institution’s capacity. The lack of skills translates into a poor labor market and problematic transition from school to work, affecting the country’s productivity ( Garcia & Fares, 2008). Varghese (2015) stated that educational cooperation is better directed to support higher education in implementing national policies and institutional enhancement.

Indonesia’s Ministry of Education and Culture increased the Darmasiswa scholarship quota from 250 to 750 students between 2015 and 2020. Darmasiswa Scholarship is a program initiated in 1974 to offer scholarships to international students from countries with diplomatic relations with Indonesia. It is a non-degree scholarship program given to international students at 51 Higher Education Institutions spread across Indonesia for 12 months. Therefore, through this scholarship program, international students can study the Indonesian language and culture at various universities in Indonesia.

*“Participants can select their subjects based on their preference, such as Indonesian language, traditional instruments (gamelan), ethnomusicology, shadow puppetry, traditional dance, crafts, the art of making batik, culinary and photography and et cetera from any of Indonesian university and college” [*Informant 2.3, Table 2 *]*

**Figure 1.**
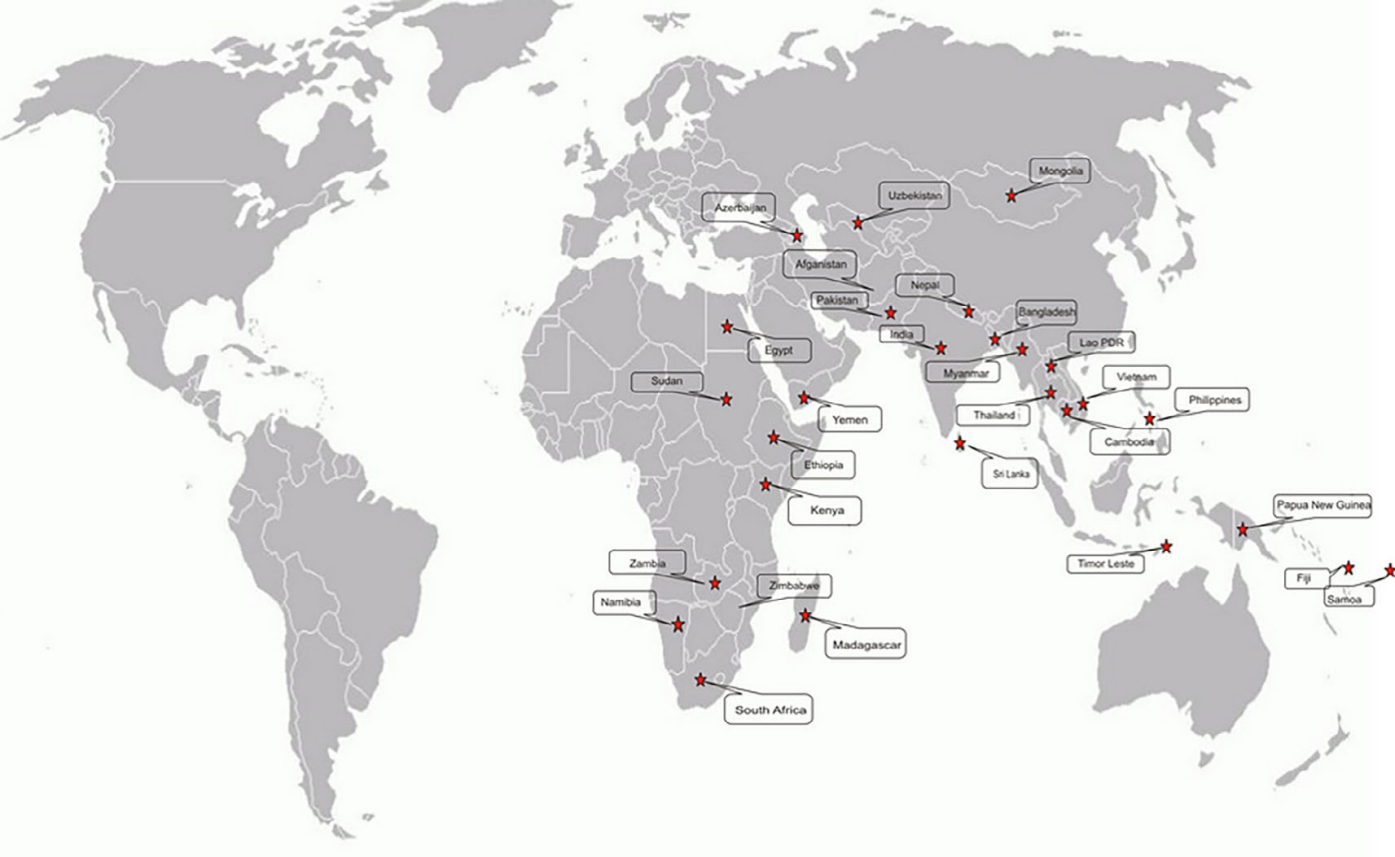
Distribution of Recipient Countries for Indonesian Assistance in the 2018 SSC Framework. Source: Ministry of Education and Culture Indonesia, 2018.

Darmasiswa Scholarship is a quota based on the Ministry of Foreign Affairs’ country’s priority, the state secretariat, and alumni ( Figure 1). The total budget allocated is IDR 40,024,025,000 covering tuition fees, living expenses, international round tickets, health insurance, and visa fees *.* Currently, there are 22 South African alumni of the scholarship program, and 4 are studying in different universities under the Darmasiswa Scholarship Program for the present academic year.

Another program is Kemendikbud Excellence Scholarships, a degree funded by the Indonesian government for international students. These scholarships are accessible to international students outside Indonesia with no limitations. It is offered to various areas of study, such as management, administration, information technology, and food security. Currently, 10 South Africans are benefitting from the Kemendikbud Excellence Scholarship. The last educational aid provided is Kemitraan Negara Berkembang (KNB), known as the *Developing Countries Partnership Scholarship* offered by the Indonesian government to prospective international students from MICs to pursue their master’s degrees. The regional exclusiveness of the scholarship was revoked in 2002 since it had developed a global interest.

Darmasiswa, Kemendikbud Excellence, and KNB Scholarship are infamous for Indonesia’s educational aid given to international students. This scholarship aims to promote Indonesian culture and language among the younger generation and strengthen cultural ties and enhance skills under the current curriculum of “ *Merdeka belajar,*” which requires international students to carry out internship programs in Indonesian industries at the end of their study. Among the three, KNB was created specifically to enhance a south-south cooperation mission in which Indonesia contributes to international public goods of education as indicated by the yearly budget allocation. However, from Indonesia’s standpoint, the scholarship is also considered charity rather than an investment for development, which is caused by the lack of framework between its SSC in education and South Africa.

Based on information from several informants, it was explained that educational cooperation between Indonesia and South Africa has so far been emphasized more on the provision of scholarships and cooperation in the field of culture. The scholarship quota has also increased from year to year. The cooperation can also be seen in the form of the MoU that has been agreed upon by the two countries. In connection with this MoU, an informant (2.2) Table 2, from the Ministry of Foreign Affairs of the Republic of Indonesia explained that:

“A Memorandum of Understanding between the Government of the Republic of South Africa was signed on March 17, 2008, in Pretoria. This agreement is valid for 5 years and will automatically be extended for another 5 years”

### The lack of SSC in the education framework

According to the Indonesia Ministry of Foreign Affairs, there is no document on the education cooperation between Indonesia and South Africa. However, universities in both countries have accidentally collaborated with South Africa’s universities, with non-state actors becoming more influential in international relations. Therefore, a proper discussion is needed to advocate bilateral relations in education at the state level, especially when honoring the SSC between MICs.

Government plays an important role in orchestrating the SSC effort between Indonesia and South Africa, from setting up funding, infrastructure, and human resources, to monitoring and evaluating the results for both sides. Most *Memoranda of Understanding* between Indonesia and overseas universities are ineffective due to the limited resources and the lack of framework. Moreover, President Jokowi mandated the Indonesia Ministry of Education and Culture to ensure all forms of an international agreement signed by Indonesian universities are registered and monitored by the state. International cooperation in the field of education needs to be ensured that it can contribute to the development of education. Therefore, it is necessary to map the cooperation that is carried out (informant 2.3) from

“In accordance with President Jokowi's directives that were mandated to the Minister of Education and Culture of the Republic of Indonesia, all forms of international cooperation in the education sector must be carried out as fully as possible, do not let cooperation only be on paper or never been implemented. Therefore, every cooperation with partner countries is reviewed by involving the relevant directorates, mapping what has been implemented and what cannot be implemented, and priority cooperation” [Informant 2.3, Table 2]

Regional exclusiveness of the Indonesian scholarship shows unexpected results and is therefore revoked. Meanwhile, inter-regional cooperation plays an integral role in advancing SSC in education and skills development. The NAASP (New Asia-Africa Strategic Partnership), a strategic inter-regional partnership between Asia and Africa, established the education cooperation that connects NAASP member countries, called the Asia-Africa Development Universities Network (AADUN), consisting of 21 universities with a few from Indonesia and South Africa.

Despite the increasing mobility of South African students to Indonesia, there is still an underlying challenge due to the state’s inadequate involvement in granting visas. While South Africans can travel to Indonesia without a visa, Indonesians do not enjoy the same benefits. They need a minimum of 30 days to get a travel visa, which is sometimes difficult to obtain. Deregulation of immigration rules between both countries can promote students, researchers, and lecturers’ relationships.

## Discussion

### South-South cooperation in education: way forward

Previous studies have shown that there is an approach of the “triangular model” in SSC ( Abdenur & Da Fonseca, 2013; Walz & Ramachandran, 2011; Sa e Silva, 2009) and the nature of the triangular model allows an effective planning by focusing on participation and long-term involvement for all parties involved. However, in this study, we argue that there is also a need to involve collaboration with civil society.

### Collaboration with civil society, NGOs, and private sectors

SSC is not a well-defined debate due to its inability to determine the role of civil society and its exclusive engagement with bilateral or state-state affairs. Interviews conducted with leaders of universities in Indonesia indicate that not many have detailed knowledge regarding the concept and practices of SSC and civil society. Rather, they mostly depend on information from the internet and social media. Civil society plays a significant role in ensuring that Indonesia’s SSC education agenda with South Africa is aligned with the needs and interests of Indonesians. Besides, as the ‘watchdogs’, the people can help identify some obstacles to meeting their various ambitions. Currently, the coordination of the civil society, NGOs, and foundation at the national level is not enough to push the agenda, as there are no concrete plans to scale up the involvement of civil society in SSC-related topics.

The process of increasing state involvement tends to develop an incentive mechanism for actors involved in South-South Cooperation, thereby promoting positive progress and outcomes. Indonesia and South Africa need to cooperate in education and skills development, especially regarding teacher training, girls’ education, halal products, and education technology by implementing the SDGs. Simultaneously, identifying requires actions for monitoring and evaluating SSC to incorporate lesson-learned into the future initiatives in education development. Therefore, with the help of civil society, NGOs, and foundations, the state can reflect on how investment-led policy platforms for SSC can further advance the implementation of the SDGs in education for both countries.

At the inter-regional level, a multilateral organization for civil society networks such as the global network of Asian African Scholars is used to promote good governance and education capable of protecting and maintaining human capacity, dignity, rights, and values. Asian-African relations are becoming increasingly important globally, in the political and economic spheres and social and cultural fields. This is because it opens up new economic development options, political solidarity, intellectual solidity, and the emergence of a transnational civil society.

There is a crucial need to engage with the academic and scientific community and leverage technical knowledge in the private sector and civil society to develop and implement practical solutions. The private sector plays a significant role in sharing information about their capacities and resources, thereby opening up opportunities to include each institution’s comparative advantages in the two countries. For example, in the current pandemic, cooperation forums with the private sector play an important role in facilitating medical equipment and medicines.

### Collaboration with universities

In 2014, joint university projects, which focus on poverty reduction and human development were established through a collaboration between the African-Asian Development University Network (AADUN), the Center for Civilizational Dialogue, the University of Malaya, the Asia Africa Option research project (AFRASO), and Goethe University Frankfurt, Germany.

Academics and higher education are significant in strengthening educational cooperation between Asia and Africa, especially in Indonesia and South Africa. Universities are becoming the front-row in international cooperation in education through the Memorandum of Understanding (MoU), which contains programs including student exchanges, research, joint scientific publications, double-degree, and training.

Since 2008, Indonesia and South Africa have been strategic partners with halal certification and education based on the Joint Declaration on Strategic Partnership. Since this partnership, several universities in South Africa have collaborated with those in Indonesia. South Africa has 26 public universities with nearly one million students in various areas, such as agriculture, mining, mineral engineering, and public health.

On 28 Jan 2020, the Chancellor of the Muhammadiyah University of Yogyakarta (UMY) and the Judicial Council (MCJ) Cape Town, South Africa, signed the MoU agreement to cooperate with the education sector through full scholarships. This program is given to South African students willing to complete undergraduate, postgraduate, and doctoral degrees. Establishing international cooperation between tertiary institutions can create superior human resources.

### Investment-Led model

A study conducted by Liao *et al.* (2019) reported a positive correlation between education investment and sustainable economic growth, with a 1% increase leading to a gross domestic product of 0.14% on average. Bhusnurmath (2016) stated that the investment-led model relies on creating new capacity, which provides more employment opportunities with higher demand and production. Therefore, any investment linked to innovation increases supply, demand, and growth. However, the influence of educational investment on economic growth is a long-run process and not immediate.

Rather than equal benefits in economic growth, a win-win in SSC means both parties have access to the output, such as a positive country’s image, transfer of knowledge, improvement in education through the investment in each other’s learning infrastructure. Others include developing the information and communications technology (ICT) and improving the quality and service of educators and facilitators. MICs often viewed science as a luxury affordable only by developed countries. Emerging countries, such as South Africa, have also managed to catch up by being Africa’s strongest scientific capacity and world-class infrastructures. The SSC promotes more mutual respect while offering substantial experience in dealing with the developing nation related-issues. Therefore, we construct a comparison between the existing model and the new model as appears in Table 3.

**Table 3.** Comparison between Existing Model of SSC in Education and investment-led Model (New Model).

	Existing Model	Investment-led (New Model)
**Nature**	Aid	Investment
**Actors**	1. Government to Government 2. Government to Southern Public 3. University to University	University, Government, Civil Society, NGO.
**Document**	Memorandum of Understanding (MoU)	Memorandum of Agreement (MoA)
**Programs**	Capacity building: 1. International students through scholarship and internship 2. Diplomat training	Capacity building for educators and trainers
		Infrastructure building in information and technology
		Health research
**Monitoring and Evaluation**	None	Time-bound

In order to achieve gradual benefit towards the contributor of SSC in Indonesia, the government needs to take adequate measures by monitoring the international student scholarship closely, adding quotas, or designing a certain priority in requirements when necessary. It rejects the concept of charity and rather emphasizes the principles of equality, respect for national sovereignty, ownership, and mutual goals.

Meanwhile, the characteristics of SSC do not require the imposition of conditions because they can strengthen the power of the irresponsible political elite at the expense of democratic reform and respect for human rights. A business-oriented approach can end unfair trade-offs; therefore, commitment needs to be ensured when designing the cooperation. There are several factors responsible for successful cooperative activities when achieving objectives. According to McKenzie *et al.* (2008), some of the key success factors of international cooperation include identifying the genuine needs of all parties, using a two-way process to share strengths to help others, and providing strong links, including central governments like ministries of education. Others include providing a program framework approved by both parties, a realistic time frame, a resourceful secretariat to maintain the implementation, including support from civil society, sharing good practices, and developing plans. The agreement must be tailored to cover both needs, and when implementing the SSC, strong political support from multi-stakeholder is required.

## Conclusion

In conclusion, the growing need and efforts to solve today’s critical problems require international cooperation and multi-stakeholder support. The cooperation agenda between Indonesia and South Africa focuses on economic balance through trading with education cooperation limited to demand-based vocational training. Furthermore, the current SSC in education between both countries is still unfathomable with the absence of resources focused on developing and implementing a south-south education cooperation model. This incidental agenda is mostly initiated and implemented by non-state actors, such as the university. This research suggests using the investment-led model involving multi-stakeholders to advance SSC in education in both countries. We recognize there were a number of limitations in this study, we sought to follow the recommendation of Du Toit (2017) to emphasize the importance of time-bound monitoring and evaluation in SSC cooperation. Nevertheless, we acknowledge that we were also not able to measure the effectiveness of the existing model of cooperation between Indonesia and South Africa. Therefore, this study suggests for future research to conduct a quantitative approach to measure the effectiveness of the model, particularly in the context of university-to-university cooperation.

## Declaration

### Author contribution statement

Dudy Heryadi: head of research, analyzed

Shanti Darmastuti: conceived, designed interview, analyzed

Ayu Anastasya Rachman: analyzed, interpreted data, wrote the paper

## Data availability

### Underlying data

Figshare: Advancing South-South Cooperation in Education: Indonesia Experience with South Africa, https://doi.org/10.6084/m9.figshare.20113892.v2 ( Heryadi *et al.,* 2022).

This project contains the following data:

- Transkrip Pertanyaan dan Jawaban Wawancara - Advancing South South Cooperation in Education Indonesia Experience with South Africa.docx
- Daftar Kerja sama Bidang Pendidikan dan Kebudayaan Negara Afrika Sub Sahara.doc
- Kerjasama Selatan-Selatan 2018.docx

Data are available under the terms of the Creative Commons Attribution 4.0 International Public License (CC-BY 4.0 International).

The transcripts of all participants will be made available in English upon request.
