# Supplementary material for: Advancing South-South cooperation in education: Indonesian experience with South Africa
Source: F1000Res. 2024 Jan 30;11:982. Originally published 2022 Aug 25. [Version 3] doi: 10.12688/f1000research.123311.3 (PMC10474336; doi:10.12688/f1000research.123311.3)
Supplement: Peer review history

# Reviewer report: Reviewer response for version 3

**DOI:** 10.5256/f1000research.157073.r242353

**Published:** 2024-02-22

**Authors:** Thomas Muhr

**Affiliations:** 1 Centre of International Studies (CEI), ISCTE-Instituto Universitario de Lisboa, Lisbon, Lisbon, Portugal

As stated in your reply to the review comments, your revised manuscript (Version 3) includes additional literature, and corresponding changes have been made by adding sentences or paragraphs and improving the structure (inserting sub-headings and shifting paragraphs around). However, most of the added literature relates to human capital theory rather than South-South cooperation (SSC) and education. According to my count, there are 12 new items in your references list, of which nine relate to human capital in their titles, one to international education, and three (all from 2013/2014) to SSC cooperation generally. Accordingly, while the section “Education as human capital” has been significantly expanded, “SSC in education” is still lacking more comprehensive and meaningful engagement with the respective (and more contemporary) literature. The remainder of your paper (pp. 7-13) is, except for a few minor modifications, identical to the previous version, with several previously raised issues unaddressed.

 In this respect, I need to draw attention to a misrepresentation of my work. On p. 5 you state:

 “Muhr (2016) explains that not only security and economy, but human capital development is also a shared responsibility of countries, “commitment to mutual support and joint efforts to achieve sustainable and integral human capital development, and the appropriate care of middle-income countries”.

 This is incorrectly cited. The correct quote cites a meaning of ‘solidarity’ in SSC:

 “solidarity being ‘The commitment to mutual support and joint efforts to achieve sustainable and integral human development, and the appropriate care of countries’ emergent needs, within the possibilities and in accordance with shared responsibilities’” (Muhr cited in Muhr, 2016:  633( Ref 1)).

 Of course, I am not disputing your inference from my work, but if you decide to cite, this has to be accurate.

 Generally, as I said in my previous comments, human development, human resources and human capital, which you seem to use interchangeably (e.g., your Table 1: title vs informants’ statements), are not (necessarily) the same, and some clarification in this respect would be useful.

 Overall, I still think your paper would benefit from a more argumentative/explanatory style to understand better how paragraphs and sections build upon each other. Regarding your approach, for me the main tension has not been resolved: why you analyse (at least partially) your SSC case study through what is widely considered a neocolonial/imperialist West/North-centric framework  – ‘human capital’, ‘aid’, International Education and ‘soft power’ (in fact, your well-chosen source, Lomer, 2017, points to the ‘colonial connotations’ of this metaphor ((also see Muhr, 2023 (Ref 2), on this)) – at least in theory, conceptually and/or discursively incompatible with the claims and objectives of SSC. I am not saying that you should not do this; but does your case – empirical findings – justify doing so? The point for me would be to make this explicit, to argue, explain, discuss, what I perceive to be a conflation of two rather opposed models. After all, transposing West/North thought (ideology) to South realities, thus reproducing coloniality, has been subject to much contention, with a growing body of literature over the past 20 years, especially since the higher education decolonisation movement from around 2015/2016.

Is the case presented with sufficient detail to be useful for teaching or other practitioners?

Partly

Is the work clearly and accurately presented and does it cite the current literature?

Partly

If applicable, is the statistical analysis and its interpretation appropriate?

Not applicable

Are all the source data underlying the results available to ensure full reproducibility?

Yes

Are the conclusions drawn adequately supported by the results?

Partly

Is the background of the case’s history and progression described in sufficient detail?

Yes

Reviewer Expertise:

Education, Development, Globalisation (Development Theory/Studies; South-South cooperation/South-South cooperation in Education; Decolonisation).

I confirm that I have read this submission and believe that I have an appropriate level of expertise to confirm that it is of an acceptable scientific standard, however I have significant reservations, as outlined above.

## References

[1] : Beyond ‘BRICS’: ten theses on South–South cooperation in the twenty-first century. *Third World Quarterly* .2016;37(4) : 10.1080/01436597.2015.1108161 630-648 10.1080/01436597.2015.1108161

[2] : South-South Cooperation. In Matthew Clarke & Xinyu (Andy) Zhao (Eds.), Elgar Encyclopedia of Development. Edward Elgar. dust. *2023;553;558* .

# Reviewer report: Reviewer response for version 2

**DOI:** 10.5256/f1000research.144425.r178210

**Published:** 2023-09-01

**Authors:** Susanne Ress

**Affiliations:** 1 University of Bamberg, Kapuzinerstraße, Bamberg, Germany

The article examines Indonesia's approach to South-South cooperation in higher education with focus on potential African partners. It argues that an investment-led model can lead to mutual benefits in human capacity development. Interviews with ministry officials and secondary data on scholarships provide the base of analysis. It concludes that a strategic approach by the Indonesian government is needed to further enhance SSC in education as a response to global challenges.

 The research topic is timely and warranted. I thank the authors for their work and congratulate on an interesting article, which I enjoyed reading very much. However, some additional revisions are needed to further enhance the quality of the argumentation. 

 Here are my suggestions:

**General comments**

- The connection between research question, review of literature (or status quo of existing research), methodology, findings and conclusions should be made clearer. How does one support the other?
- More references are needed to substantiate claims throughout the article (e.g., 2 ^nd^ paragraph on page 5 of the pdf)
- I suggest using “low-, middle-, high-income” countries as classification, use classification consistently
- Check full article for correct use of quotation marks and provide appropriate reference, including page numbers
- Carefully proof-read the article for punctuation, grammar, missing words

**Introduction**

- Cite relevant literature on south-south cooperation in education (suggestions see below)
- The introduction would benefit from a more nuanced understanding of international development education concepts and approaches
- Provide references to the literature for information on context
- Explain the context of the study in greater detail, for example, explain the Indonesian SSC strategy in relation to “Merdeka Belajar” – how are the two related in the Indonesian education landscape? Has the Indonesian government developed a SSC strategy based on “Merdeka Belajar” or do interview respondents refer to it as justification for their internationalization efforts?
- Please include a clearly stated research question in the introduction
- P. 3, 5 ^th^ paragraph “It is aimed to advocate FOR the academic …” – word missing
- P. 4, 1 ^st^ paragraph – correct use of quotation marks, provide page numbers and reference to source (check the article throughout)
- P. 4, 2 ^nd^ paragraph – “… several studies …” – please provide references

**Literature review**

*Education as human capital*

- I suggest considering additional conceptual frameworks, for example “soft power” (to explain political function of scholarships, see findings p. 8, Darmasiswa Scholarship) or “education policy transfer”
- Explain the investment-led model in greater detail, how is it different from other models? Perhaps some of the discussion could move here.

*SSC in education*

- P. 5, 2 ^nd^ paragraph – text is repeated from previous paragraphs, provide references to the literature
- P. 6, 1 ^st^ paragraph, last sentence – word is missing “Therefore, IN our model these two …”
- Please explain your use of “more progressive models” – what do you mean by progressive?
- Please explain “triangular model” in greater detail since you refer to it in the discussion. The explanation given in the discussion is also not sufficient.
- Overall, the literature review would benefit from a more analytical structure rather than an additive summary of articles. One possible structure could be: a) actors of SSC, b) modes of SSC, c) benefits of SSC, d) challenges of SSC in education. This would provide an opportunity to incorporate existing research on SSC in education more broadly (suggestions for additional authors: Abdenur, Chrisholm, Cesarino, Majee, Morais, Ress, Soudien).
- Overall, the literature review would benefit from a more nuanced understanding of human capital theory, including past and present criticisms (e.g., Bowles & Gintis, 1975; Gillies, 2015).

**Methods**

- P. 6, 2 ^nd^ paragraph – correct “Cresswell & Cresswell
- Please justify the selection of organizations for interviews based on the research question.

**Findings**

- P. 8, last paragraph – use of the word “infamous” – consider revising since it seems contradictory

**Discussion**

- It is missing the connection to the findings, for example add quotes
- Explain the investment-led model as part of the literature review
- Explain more clearly how the findings of the study support the investment-led model (or not)
- Focus on how your data (interviews and scholarship data) support the investment-led model (or not)
- Provide a more nuanced understanding of the benefits and especially the challenges of SSC in (higher education)

Is the case presented with sufficient detail to be useful for teaching or other practitioners?

No

Is the work clearly and accurately presented and does it cite the current literature?

Partly

If applicable, is the statistical analysis and its interpretation appropriate?

Not applicable

Are all the source data underlying the results available to ensure full reproducibility?

Partly

Are the conclusions drawn adequately supported by the results?

Partly

Is the background of the case’s history and progression described in sufficient detail?

Partly

Reviewer Expertise:

Internationalization of higher education, south-south and north-south cooperation in higher education, comparative and international education

I confirm that I have read this submission and believe that I have an appropriate level of expertise to confirm that it is of an acceptable scientific standard, however I have significant reservations, as outlined above.

## References

[1] : ‘Introduction: Rhetorics, Realities, and Responses’, in L. Chisholm and G. Steiner-Khamsi (eds), South–South Cooperation in Education and Development. *New York: Teachers College.* .2009;1-13

[2] : Tilting the North-South Axis: The legitimization of Southern Development Knowledge and its Implications for Comparative Education Research. *Current Issues in Comparative Education* .2002;4(2) : 10.52214/cice.v4i2.11356 10.52214/cice.v4i2.11356

[3] : Organisation and Politics in South–South Cooperation: Brazil's Technical Cooperation in Africa. *Global Society* .2015;29(3) : 10.1080/13600826.2015.1033384 321-338 10.1080/13600826.2015.1033384

[4] : South−South cooperation and democracy in Africa: Brazil’s role in Guinea-Bissau. *Africa Review* .2013;5(2) : 10.1080/09744053.2013.855356 104-117 10.1080/09744053.2013.855356

[5] : South–South cooperation across the Atlantic: Emerging interfaces in international development and technology transfer in agriculture’ Dissertation. *University of California, Berkley* .2013;

[6] : Colonial legacies in internationalisation of higher education: racial justice and geopolitical redress in South Africa and Brazil. *Compare: A Journal of Comparative and International Education* .2020;50(4) : 10.1080/03057925.2018.1521264 463-481 10.1080/03057925.2018.1521264

[7] : Internationalization of Higher Education for Development. Blackness and Postcolonial Solidarity in Africa-Brazil Relations. New Directions in Comparative and International Education Series. *Bloomsbury Publishing* .2019;

[8] : South–South cooperation, policy transfer and best-practice reasoning: The transfer of the solidarity in literacy program from Brazil to Mozambique. *ISS Working Paper Series/General Series* .2005;1-57

[9] : Evaluation of sowing dates for managing yellow mosaic disease caused by mungbean yellow mosaic India virus in mungbean. *3 Biotech* .2023;13(6) : 10.1007/s13205-023-03621-z 207 10.1007/s13205-023-03621-z 37229276 PMC10203070

[10] : The problem with human capital theory--a Marxian critique. *The American Economic Review* .1975;2(65) :74-82

[11] : India and South-Africa: Diaspora and Transfer’, in L. Chisholm and G. Steiner-Khamsi (eds), South–South Cooperation in Education and Development. *New York: Teachers College* .2009;

# Reviewer report: Reviewer response for version 2

**DOI:** 10.5256/f1000research.144425.r178212

**Published:** 2023-07-14

**Authors:** Thomas Muhr

**Affiliations:** 1 Centre of International Studies (CEI), ISCTE-Instituto Universitario de Lisboa, Lisbon, Lisbon, Portugal

Thank you for inviting me to review this article. Entering a review process at a later stage and picking up from previous reviewers is a challenge for both reviewers and authors. I aim to practice fairness by taking the previous reviewer Indra Yohanes Kiling’s (henceforth abbreviated IYK) comments and decision as a benchmark, to which I add my own thoughts. I address you – the authors – directly. 

 I enjoyed reading this over large parts informative, research-based piece, guided by a strong rationale as stated in the final lines of paragraph 6 on p. 3 (first page of the Introduction, ‘Although this collaboration...’). Also, the objective is clearly stated (end of first paragraph of the Introduction; re IYK’s query). The empirical focus on Indonesia/South Africa is an extremely useful and welcome case study to contribute to the relatively scarce literature on South-South cooperation (SSC) in education. The main message I get from your paper is a call for the neoliberalization or (in Walter Mignolo’s terms) ‘re-Westernization’ of SSC, which to me does not sit easily with the general raison d´être of SSC and the current historical development of the South’s growing de-linking (e.g., de-dollarizing intra-South trade, growing South-South student mobility/flows). With this in mind, as IYK suggested (to which you have not responded), providing some clarification/discussion of your positioning/positionality in this study would increase trustworthiness. My subsequent detailed comments largely relate to these broad issues.

 1. Re IYK’s comments on the literature used and conceptual limitations: You have followed IYK’s suggestion to integrate more recent literature (Hanada, 2021; Nguyen et al, 2015; Zhu & Chikwa, 2021). Two of these (Hanada, 2021; Nguyen et al, 2015), however, are in the field of conventional International Education, i.e. collaborations between institutions in the North and South. This practice (also sometimes associated with triangular cooperation), however, is often critiqued for being North/Western-centric, to pull Southern institutions into the circuits of the North-driven neoliberal global higher education governance (a mechanism of ‘re-Westernisation’, domination and control of the South). This is not SSC. So the newly added literature is of only limited relevance to your topic because your Indonesia/South Africa case clearly is South-South, not North-South or triangular cooperation. As recommended by IYK, you need to consult more specific education SSC literature, including reviews of any sort. I consider this necessary for the following two interrelated reasons:

- On one hand, I perceive a conflation of Northern ‘aid’ (official development assistance, ODA) discourses/concepts with SSC ones. For example, you seem to associate SSC with ‘middle income countries’, ‘donors’, ‘charity’, and ‘comparative advantage’. At the same time you also use ‘global south’, ‘partners’ and ‘win-win’, which is more like the language SSC actors commonly use. Certainly, there are hybrid discourses (e.g. to some extent in BRICS). If this is the case in Indonesia/South Africa SSC, this would be a relevant finding and would have to be discussed as such, i.e. explaining why you appear to be using ideas and concepts from these different – rather incompatible – political projects interchangeably.
- On the other hand, you are using human capital theory to frame SSC in education, which is not very common in this field. Did you come across relevant SSC education literature that uses human capital theory? (I am referring here really to SSC literature and not North-South International Education literature). Does the human capital framework relate to or emerge from your empirical (interviews or documentary) research, i.e. do the Indonesian/South African actors themselves refer to this? If so, it would be important to make this explicit. If not, if this framing is purely your choice (considering that you are referring to a ‘deductive’ approach without further explaining this, p. 6, para 6), however, I wonder why conduct empirical research if this is squeezed into a pre-established rather Western-centric, colonialist theoretical/conceptual framework (of which ‘human capital theory’ is part). In fact, your Table 1 suggests that while your interviewees themselves refer to ‘human resources’ (which also appears in SSC discourses I am familiar with), your title of the table appears to equate this with ‘human capital’, i.e., you seem to use the two interchangeably. But ‘human resources’ is not the same as human capital (theory). Nor is ‘human development’, which you also use in these contexts (‘human development’ as commonly associated with the 1980s UN project of conceiving a liberal-egalitarian, equality-driven challenge to economistic reductionism, of which human capital theory forms part).

These conceptual ambiguities need to be clarified in one way or another. Also, regarding concepts, you refer to ‘conditions’ (p. 11, para. 6), but the appropriate term is ‘conditionality’/‘conditionalities’.

 2. IYK raised some methodological issues you have responded to. I have some minor remarks in this regard: Throughout, including the Abstract, frequent reference is made to ‘an interview’ and ‘the interview’. I could not detect a clear statement of how many interviews were actually conducted and analysed, although you do state that ‘nine people’ (p. 6, para 4) participated as informants (so I assume 9 interviews were conducted?). It would be useful to clearly state from the start (Abstract/Introduction) the number of interviews conducted, perhaps also how long they were on average and whether an interview guide was used (e.g., with respect to structured, semi-structured or conversational interviews, or any other type). Also, it would be helpful for the reader to know the exact research questions (which you point to on p. 6, para. 2), and when (year/months) the research was conducted (re ‘The data were obtained within six months’, as stated in the Abstract and p. 6, para. 5).

 3. IYK suggested adding more quotes from your interviews. I am not sure whether all the quotes you have inserted really add a lot. Perhaps re-check your transcripts for particularly meaningful quotes. 

 4. There is a factual error: OPEC is not part of the ‘traditional development aid’ architecture (p. 5, para. 5). OPEC was formed in Baghdad in 1960 within the SSC logic. This needs correcting.

 5. I sometimes perceive vagueness and questionable evidence, e.g.: ‘Dr. Diplomat in the Economist mentioned a study...’ (p. 5, para. 5); ‘there is so much theory’ (p. 5, para. 6); ‘more progressive models, like investment-led mode (p. 6, para. 1; Question: what is progressive about investment-led modes?)

 6. The section ‘Discussion’ (pp. 10-11) reads more like a bullet-point-type list of recommendations, whereby it is not always clear how these rather normative statements/claims were generated from the data presented in the previous section.

 7. Some minor linguistic, stylistic and structural issues/suggestions:

1. The overall quality of your article is undermined by quite a few little linguistic errors or imprecisions (often related to a – in my opinion – inappropriate use of the definite article). This concerns the entire paper. Just a few examples: in the first paragraph of the Introduction, I believe ‘Compared to the North-South Cooperation, the South-South is perceived...’ should read ‘Compared to North-South Cooperation, South-South cooperation is perceived...’; in paragraph 3 on the same page, ‘from other country’ should be ‘other countries’; or, ‘countries have accidentally collaborated’ (p. 9 para. 4; I am not sure whether ‘accidentally’ is the most appropriate verb here).
2. There are a couple of incomplete citations (missing reference/page numbers).
3. There are occasionally statements and paragraphs that to me do not make a lot of sense in the context/section in which they appear. E.g., reference to USA cooperation with Africa (North/South cooperation) in the section ‘SSC in education’ (p. 5, para. 3; again mixing the two paradigms); para. 1 on p. 8 and para. 2 on p. 11 appear misplaced and could be integrated in previous sections.
4. Please address the inconsistencies regarding capitalizations in South-South cooperation: you interchangeably use ‘South-South cooperation’, ‘South-South Cooperation’, ‘south-south cooperation’, or abbreviated ‘SSC’ (unless you seek to highlight different meanings attached to each of these, which however would need explaining).
5. The ‘Methods’ section clearly states the two conceptual frameworks used, i.e. ‘Education and Human Capital’ and ‘South-South cooperation Model’/‘South-South Cooperation framework’. I propose to already state these in the Abstract and Introduction. The article would benefit from - requires - clearer discussion of how the two relate to each other (my Point 1 above).
6. An apparent inconsistency/contradiction: p. 3 para. 2 points to ‘non-interference’ as a key feature of SSC, while on p. 7 para. 5 the paper calls for a ‘business-oriented approach’ to SSC to ‘avoid interference’. In this context, the Western (neoliberal) ‘good governance’ conditionalities of ‘democratic reform’ and ‘human rights’ pop up (p. 7, para. 5; p. 11, para. 6), however, none of this was discussed/introduced previously. (This relates to my initially-stated impression of your paper seeking to promote a ‘re-Westernisation’ of SSC – or a re-definition of the SSC principles? Is this your intention or positioning?)
7. The newly added paragraphs in the Introduction make this a little bit long and bulky. Perhaps some of this would better be integrated in the next section, Literature Review.

Overall, I find the tone of your paper too assertive and proclamatory, and at times undersubstantiated. There is very little explaining, arguing and discussing. I propose careful and judicious editing, taking the issues raised into account.

Is the case presented with sufficient detail to be useful for teaching or other practitioners?

Partly

Is the work clearly and accurately presented and does it cite the current literature?

Partly

If applicable, is the statistical analysis and its interpretation appropriate?

Not applicable

Are all the source data underlying the results available to ensure full reproducibility?

Yes

Are the conclusions drawn adequately supported by the results?

Partly

Is the background of the case’s history and progression described in sufficient detail?

Yes

Reviewer Expertise:

Education, Development, Globalisation (Development Theory/Studies; South-South cooperation/South-South cooperation in Education; Decolonisation).

I confirm that I have read this submission and believe that I have an appropriate level of expertise to confirm that it is of an acceptable scientific standard, however I have significant reservations, as outlined above.

# Reviewer report: Reviewer response for version 2

**DOI:** 10.5256/f1000research.144425.r163939

**Published:** 2023-02-24

**Authors:** Indra Yohanes Kiling

**Affiliations:** 1 Faculty of Public Health, Universitas Nusa Cendana, Kupang, Indonesia

Thank you to the authors for their commitment in revising this article. I think this article is now worthy to be indexed. Congratulations to the authors and good luck for their endeavours!

Is the case presented with sufficient detail to be useful for teaching or other practitioners?

Yes

Is the work clearly and accurately presented and does it cite the current literature?

Partly

If applicable, is the statistical analysis and its interpretation appropriate?

Not applicable

Are all the source data underlying the results available to ensure full reproducibility?

Yes

Are the conclusions drawn adequately supported by the results?

Yes

Is the background of the case’s history and progression described in sufficient detail?

Yes

Reviewer Expertise:

Community psychology, qualitative studies

I confirm that I have read this submission and believe that I have an appropriate level of expertise to confirm that it is of an acceptable scientific standard.

# Reviewer report: Reviewer response for version 1

**DOI:** 10.5256/f1000research.135406.r162006

**Published:** 2023-02-06

**Authors:** Indra Yohanes Kiling

**Affiliations:** 1 Faculty of Public Health, Universitas Nusa Cendana, Kupang, Indonesia

This is a well-written article. It has the potential to advance current literature and practice in the field of education. I have some suggestions to the authors to help improve the article.

- The introduction needs more work especially in citing the more recent literature. I would like to suggest the authors to find some relevant systematic or scoping reviews to help in highlighting the current evidence and research gap. I think the authors could also work on making the objectives of the study more explicit.
- The authors should discuss their positioning as a research team in this study. This kind of reflexivity will help to improve trustworthiness of the study.
- Moreover, I would suggest the authors use other efforts to improve trustworthiness of the study besides using triangulation. I would like to recommend the authors to at least use audit trail and diary which is vital to any qualitative study. If they have these documents, they should attach them as appendixes.
- Some more quotes can be used to help explain themes.
- The discussion is strong in the contextual and implications but I think is still lacking in terms of conceptually. Some analysis on the limitations and strengths of the study might benefit the readers as well.

Is the case presented with sufficient detail to be useful for teaching or other practitioners?

Yes

Is the work clearly and accurately presented and does it cite the current literature?

Partly

If applicable, is the statistical analysis and its interpretation appropriate?

Not applicable

Are all the source data underlying the results available to ensure full reproducibility?

Yes

Are the conclusions drawn adequately supported by the results?

Yes

Is the background of the case’s history and progression described in sufficient detail?

Yes

Reviewer Expertise:

Community psychology, qualitative studies

I confirm that I have read this submission and believe that I have an appropriate level of expertise to confirm that it is of an acceptable scientific standard, however I have significant reservations, as outlined above.

# Response

**Published:** 2023-02-14

**Authors:** Dudy Heryadi

**Affiliations:** Universitas Pembangunan Nasional Veteran Jakarta, Indonesia

Dear Dr. Indra, thank you for your review. We have looked through it and agree to make changes accordingly to improve the article in order to advance current literature and practice in the field of education.

# Response

**Published:** 2023-02-14

**Authors:** Dudy Heryadi

**Affiliations:** Universitas Pembangunan Nasional Veteran Jakarta, Indonesia

Dear reviewer, here are point-by-point respond made according to the given comments.

1. Comment: *"The introduction needs more work especially in citing the more recent literature. I would like to suggest the authors to find some relevant systematic or scoping reviews to help in highlighting the current evidence and research gap. The authors should discuss their positioning as a research team in this study. This kind of reflexivity will help to improve trustworthiness of the study." *
**Respond: The new version have cited and discussed more recent literature by Nguyen et al. (2015), Hanada (2021), and Zhu & Chikwa (2021)**
2. Comment : *"the authors could also work on making the objectives of the study more explicit."*
**Respond: The new version have highlighted research gap and study the objectives based on the research gap founded**
3. Comment: " *to improve trustworthiness, recommend the authors to at least use audit trail and diary, they should attach them as appendixes."*
**Respond: Based on data disclose policy from F1000 is already made available through Figshare and it is stated in the appendix. The reader may refer to the following link https://doi.org/10.6084/m9.figshare.20113892.v2 ( Heryadi *et al.,* 2022).**
4. Comment: " *More quotes can be used to help explain themes." *
**Respond: The new version have added more quotes to explain the themes of international education cooperation (role and the rationale of the government)**
5. Comment: *"Lacking in terms of conceptually. Some analysis on the limitations and strengths of the study might benefit the readers as well."*
**Respond: The new version have added and highlighted the limitation and strength in terms of conceptually. **

Thank you so much for your valuable inputs.
